# Covalently Binding Atomically Designed Au_9_ Clusters to Chemically Modified Graphene**

**DOI:** 10.1002/anie.201504334

**Published:** 2015-07-06

**Authors:** Concha Bosch-Navarro, Zachary P L Laker, Helen R Thomas, Alexander J Marsden, Jeremy Sloan, Neil R Wilson, Jonathan P Rourke

**Affiliations:** Department of Physics, University of Warwick Coventry, CV4 7AL (UK); Department of Chemistry, University of Warwick Coventry, CV4 7AL (UK) E-mail: j.rourke@warwick.ac.uk

**Keywords:** gold nanoparticles, graphene, graphene oxide, nanoclusters, thiols

## Abstract

Atomic-resolution transmission electron microscopy was used to identify individual Au_9_ clusters on a sulfur-functionalized graphene surface. The clusters were preformed in solution and covalently attached to the surface without any dispersion or aggregation. Comparison of the experimental images with simulations allowed the rotational motion, without lateral displacement, of individual clusters to be discerned, thereby demonstrating a robust covalent attachment of intact clusters to the graphene surface.

Gold nanoparticles (Au NPs) have structure- and size-dependent optical and electronic properties,[1–4] and their catalytic activity increases when the particle size drops down to around 1 nm.[5] To achieve this size, an accurate design of ligand-protected gold nanoclusters (Au NCs) is required. A family of phosphine-coordinated Au NCs ([Au_*n*_L_*m*_]^*z*+^; *n*=1–11, *z*=1–4; L=PPh_3_ or PPh_2_(CH_2_)_3_PPh_2_) having well-defined nuclearity and geometrical structures have been synthesized[6, 7] and shown to exhibit clearly distinguishable optical and electronic properties.[7–10] However, the immobilization of Au NCs onto a support is an important step towards their implementation in practical devices, which has so far not been realized satisfactorily.[1, 2, 11, 12] Therefore, the challenge is to develop a strategy to chemically bind predesigned Au NCs onto the surface of solid conductors/semiconductors, thus allowing direct correlation between property and structural features.

Graphene (G), as a two-dimensional system with outstanding electronic properties and a high surface area,[13] offers the ideal platform for the deposition of NPs.[14] In addition, the diversity of carbon chemistry offers many routes to producing chemically modified graphene (CMG).[13, 15, 16] Nanoparticles have been stably attached to both G and CMGs for a plethora of different applications.[17] In particular, different routes for the hybridization of Au NPs with G have been studied,[18–23] but surprisingly, the fabrication of atomically precise Au NCs supported on G remains unexplored. Recently, we have described an easy way to chemically modify graphene with sulfur functionalities,[24] and now, by taking advantage of the affinity between gold and sulfur, we describe the stable attachment of preformed [Au_9_(PPh_3_)_8_](NO_3_) clusters[25] to our CMG. Aberration-corrected transmission electron microscopy (ac-TEM) has been employed to directly identify individual covalently attached Au_9_ clusters, and to track their relative orientation.

Chemically modified graphene with sulfur functionalities was synthesized by treatment of graphene oxide (GO) with potassium thioacetate, followed by an aqueous work-up.[24] This route is simple and scalable, and gives a single-layer material with reactive thiol groups that offer anchoring points for further functionalization; this material is referred to as GOSH. Moreover, the synthetic route results in a partial reduction of the GO when the sulfur functionalities are introduced, thereby giving a more graphene-like substance; this is particularly relevant for applications in which a semiconducting/conducting behavior is required, as the reduction of GO results in a partial restoration of the sp^2^ structure of G.[26]

The [Au_9_(PPh_3_)_8_](NO_3_) cluster (abbreviated as Au_9_) was selected as the target cluster.[10, 25] The *D*_2*h*_-symmetric cluster is composed of nine gold atoms arranged such that one central gold atom is surrounded by the remaining eight gold atoms, each of which is coordinated by a monodentate phosphine ligand (see Figures S1 and S2 in the Supporting Information). The average metal–metal distance is around 0.27 nm, which results in a cluster diameter between 0.45 nm and 0.54 nm,[10] far smaller than that typically exhibited by Au NPs (particle size >3 nm).[11] The binding between Au_9_ and GOSH was achieved by simply stirring Au_9_ with a dispersion of GOSH (Scheme 1). A covalent bond is formed between sulfur and gold, which is accompanied by displacement of a phosphine ligand. As a result, a neutral GOSH@Au_9_ hybrid is formed.

**Scheme 1:**
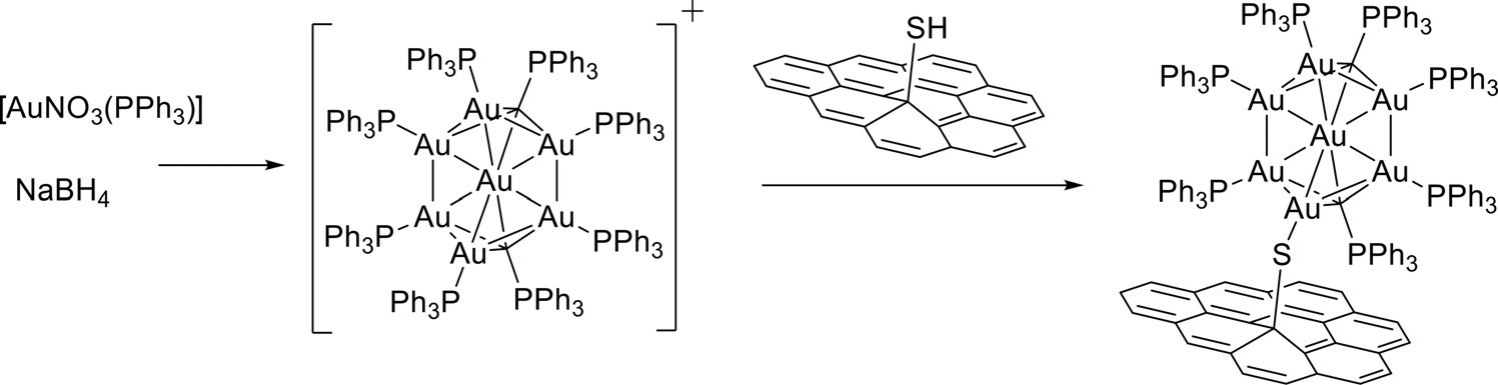
Synthesis of GOSH@Au_9_.

A comparison of the thermogravimetric analysis (TGA) results for GOSH and GOSH@Au_9_ gives the first evidence of hybrid formation (Figure S7). In both cases, a first weight loss appears around 130 °C, which corresponds to desorption of water, while the major mass loss is above 500 °C and comes from decomposition of graphene-like sheets. The main difference appears at higher temperatures, where a mass of 10.5 % remains for GOSH@Au_9_, thus pointing to the presence of metallic centers at a level of order of 1 atomic %. The presence of gold in the hybrid was confirmed by energy-dispersive X-ray (EDX) elemental analysis, which showed atomic gold and phosphorus contents of around 1 atomic % for each element. Additional, and more accurate, corroboration came from X-ray photoelectron spectroscopy (XPS; Figures S4–S6), in which, in addition to the signals corresponding to C, S, and O that are typical of GOSH,[24] signals for Au and P were found, consistent with the presence of Au_9_.[6] Moreover, the ratio between the atomic content determined by XPS (1.6 % Au:1.3 % P) is consistent with the presence of Au_9_P_7_ clusters.

A control experiment was performed whereby, instead of GOSH, GO was treated with Au_9_. With GO, no gold was detected by either EDX or by XPS (Figure S5), thus ruling out the possibility of an interaction between Au_9_ and any remaining oxygen functionalities on the GOSH, thus confirming that Au_9_ attachment is through the sulfur functionalities.

Determining where the Au_9_ is attached and whether the clusters remain intact requires direct imaging of the GOSH@Au_9_ hybrid at atomic resolution. This was achieved by ac-TEM. Atomically thin 2D materials such as graphene or graphene oxide have been employed as supports for imaging molecular species;[27, 28] at one atom thickness of carbon, they are almost transparent under the electron beam so that individual molecules can be resolved at atomic resolution, whilst their well-defined crystal lattice enables accurate calibration for quantitative measurements. TEM grids were prepared by adding one drop of a well-dispersed 0.08 mg mL^−1^ solution of GOSH@Au_9_ in DMSO to a lacey carbon support and allowing it to dry under ambient conditions.

An ac-TEM image of a typical area of GOSH@Au_9_ is shown in Figure 1 a. As is characteristic for samples derived from graphene oxide,[29] the image shows ordered regions where the graphene-like lattice is visible, regions of higher contrast that are apparently disordered and can be attributed to oxidation debris[30, 31] or other carbonaceous material adhered to the surface, defects, and small holes. A single hexagon of spots can be seen in the inset diffraction pattern, which shows that the long-range crystalline order of graphene is retained, and that this region consists of a single monolayer of chemically modified graphene (see also Figure S8).[27] Additional features are seen, not observed on GO or GOSH. Clusters of dark spots indicative of atoms with a high atomic number are apparent and dispersed across the GOSH surface. There is clear structure within the clusters and, as discussed below, this can be used to unambiguously identify these features as Au_9_ clusters. No aggregation of the clusters was observed, with isolated clusters distributed uniformly across the sheets (Figure S9b), thus suggesting that they are attached to the GOSH and hence unable to diffuse and coalesce.

**Figure 1:**
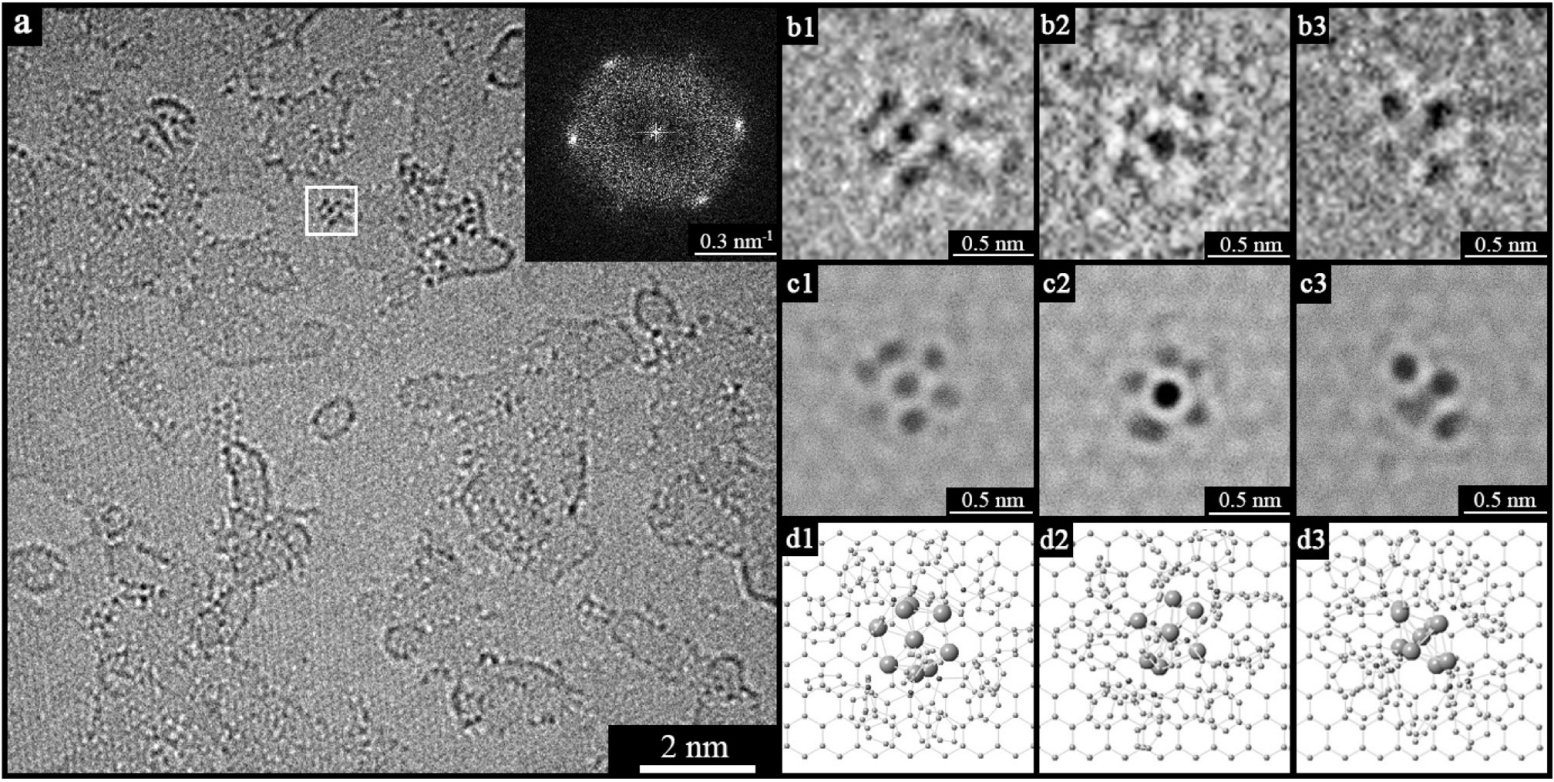
a) General ac-TEM image of GOSH@Au_9_. Inset: the typical hexagonal pattern of a graphene monolayer. b) Three different ac-TEM images with high resolution for Au_9_ imaged over GOSH in three different orientations. b1 corresponds to the molecular cluster highlighted with a square in (a). c) ac-TEM image simulation produced from the crystal-structure model depicted in (d).

Counting the Au_9_ clusters gives an estimate of the extent of functionalization. From analysis of images such as Figure 1 a, the estimated concentration of Au_9_ clusters is 7±2 per 100 nm^2^, which corresponds to just under 0.2 % of the carbon atoms in GOSH being functionalized through the sulfur to Au_9_ linkage. Note that this is only an estimate of the Au_9_ cluster density, as the image contrast of the Au_9_ clusters varies considerably depending on their orientation (as discussed below), which complicates their identification. However, this functionalization density would correspond to an atomic content of 1.6±0.4 % Au, which is in close agreement with the TGA, XPS, and EDX measurements.

More detailed analysis requires comparison between the experimental images and image simulations of the Au_9_ clusters on GOSH. Using the known crystal structure of Au_9_, a tableau of multislice image simulations was constructed by rotating the molecule about two orthogonal symmetry axes (Figure S10). For Au_9_, the contrast is dominated by the gold atoms (*Z*=79), which scatter the electrons to a greater extent than do the phosphorus (*Z*=15) or carbon (*Z*=6) atoms. However, for accurate comparison all atoms were contained in the image simulation, including the coordination sphere of ligands and a section of graphene lattice.[32] Figure 1 b shows three different regions on the GOSH@Au_9_, each from an ac-TEM image acquired with 0.3 s exposure. The region in Figure 1 b1 is marked by the box in Figure 1 a. A comparison with the simulations (Figure 1 c) facilitates identification of the orientation of the Au_9_ clusters (Figure 1 d).

Comparison between the image simulations and experimental images clearly shows that the clusters are Au_9_, with a close match for the contrast arising from the gold atoms and also subtler variations in contrast that are consistent with the ligands still being present. Further confirmation comes from the measurement of the spacings of the atomic columns within the clusters, which shows that the Au–Au distances in the experimental images are consistent with those expected for Au_9_ (Figure S11). This proves that intact, undamaged, Au_9_ NCs are present on the GOSH surface.

A study of the dynamics of these Au_9_ clusters demonstrates that they are covalently bound to GOSH and not simply adsorbed to the surface. The ac-TEM image shown in Figure 1 a was taken with a 0.3 s exposure; the match between the experimentally observed contrast and image simulation shows not only that Au_9_ is present and intact, but also that those clusters are stationary on the GOSH surface for the period of that exposure. However, inspection of subsequent images shows that the clusters are not permanently fixed and their contrast changes over time. Figure 2 shows a sequence of ac-TEM images of a single Au_9_ cluster on GOSH; images were acquired at 0.3 s intervals over a period of more than 10 s (the full image sequence is shown in Figure S12 in the Supporting Information, with only selected images shown in Figure 2). The images are from the same region and, through comparison with fixed points in the larger image (Figure S9a), show no apparent lateral displacement of the cluster relative to the underlying GOSH. The change in contrast is indicative of rotations of the Au_9_ cluster relative to the GOSH surface, and comparison with the image simulation tableau enables each of the images shown in Figure 2 to be identified as specific Au_9_ orientations. From this it is apparent that the Au_9_ cluster is rotating, but without lateral displacement. This is consistent with covalent attachment of Au_9_ to GOSH through the –S–Au bond.

**Figure 2:**
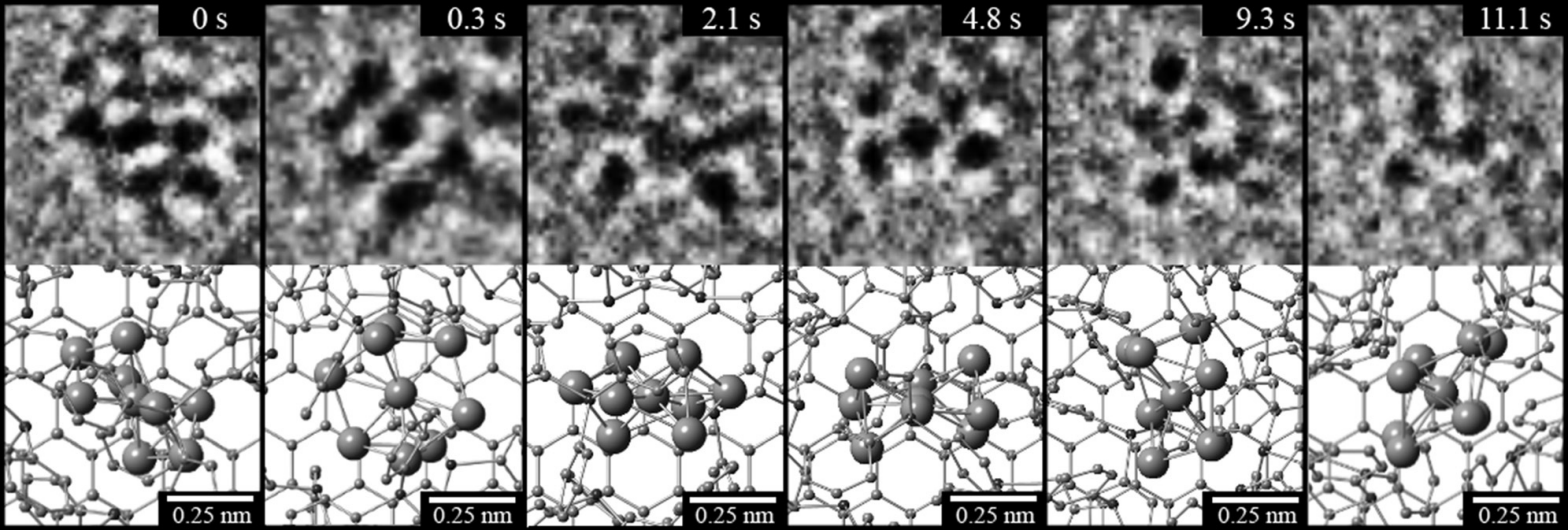
Selected ac-TEM image frames taken from the dynamics of a single Au_9_ with their corresponding molecular models (the full ac-TEM sequence representing the motion of a single Au_9_ cluster over a period of 11.4 s is shown in Figure S12).

The inhibited rotation is caused by interaction with the electron beam and is indicative of a set of metastable orientations. Prior work has observed similar electron beam induced molecular motion on graphene oxide,[32] on carbon nanotubes,[33, 34] and for molecules attached to carbon nanohorns, where it was shown that lower acceleration voltages in the TEM resulted in higher frequency of molecular motion as a result of a larger scattering cross-section.[35, 36] An accelerating voltage of 80 kV was used here to minimize damage to the chemically modified graphene by the electron beam. At this acceleration voltage, the clusters are fixed in each orientation for timescales on the order of seconds before switching to another orientation. This indicates that each observed orientation is metastable, and corresponds to a local energy minimum. As each image shows well-defined spots rather than blurred streaks, it is also clear that the transition between orientations must be relatively rapid.

In conclusion, we have proven that atomically designed clusters can be covalently attached to chemically modified graphene by taking advantage of the affinity between gold and the sulfur functionalities present on the surface. We have demonstrated that the Au_9_ clusters are intact and well-dispersed over GOSH, and show no apparent aggregation. Dynamic ac-TEM measurements show how a single molecular cluster rotates as a result of the effect of the electron beam, but without lateral diffusion, which is indicative of a strong covalent interaction between Au_9_ and GOSH. Moreover, the results of the dynamic study suggest the presence of metastable orientations that may appear as a consequence of the steric demands of the ligands.

Our approach is generally applicable to the whole family of gold nanoclusters, and may be extended to other atomically designed clusters. This will allow fine-tuning of the graphene–nanocluster properties (e.g. optical or charge-transfer properties), thus permitting exploration of the effect that the size and morphology of clusters have in applications ranging from biosensors or biomedicine, to energy storage, or heterogeneous catalysis.

## Supporting Information

As a service to our authors and readers, this journal provides supporting information supplied by the authors. Such materials are peer reviewed and may be re-organized for online delivery, but are not copy-edited or typeset. Technical support issues arising from supporting information (other than missing files) should be addressed to the authors.

gif_stack

miscellaneous_information
