# Supplementary material for: Covalently Binding Atomically Designed Au9 Clusters to Chemically Modified Graphene
Source: Angew Chem Int Ed Engl. 2015 Jul 6;54(33):9560–3. doi: 10.1002/anie.201504334 (PMC4539594; doi:10.1002/anie.201504334)
Supplement: Supplementary file 2 — miscellaneous_information [file anie0054-9560-sd2.pdf]

Angewandte Chemie
Eine Zeitschrift der Gesellschaft Deutscher Chemiker

Supporting Information

Covalently Binding Atomically Designed Au_9 Clusters to Chemically Modified Graphene**

Concha Bosch-Navarro, Zachary P. L. Laker, Helen R. Thomas, Alexander J. Marsden, Jeremy Sloan, Neil R. Wilson,* and Jonathan P. Rourke**

anie_201504334_sm_miscellaneous_information.pdf
anie_201504334_sm_GIF_STACK.avi

Supporting Information

Synthetic protocol 2

Crystal structure, distances, and symmetry relationships in $[Au_9(PPh_3)_8](NO_3)$. 3

Raman spectra 4

XPS spectra and EDX data 5

Thermogravimetric analysis of GOSH and GOSH@Au_9. 8

TEM image and XRD pattern 9

Image simulation tableaux 11

Metal-Metal distances extracted from ac-TEM images 13

Study of the dynamics 15

Synthetic Procedure

Synthesis of [$Au_9(PPh_3)_8$](NO_3) clusters (Au_9). [1] To obtain Au_9 a three step procedure was followed. First, PPh_3 (300 mg) was dissolved in ethanol (10 mL), followed by the addition of $HAuCl_4$ $3H_2O$ (200 mg) in ethanol (1 mL). The mixture was stirred for 10 minutes until a white precipitate appeared. After filtering under vacuum and washing with ethanol, PPh_3AuCl was obtained (^{31}P-NMR: 33.41 ppm). In a second step, to a solution of PPh_3AuCl (160 mg) in ethanol (1 mL), $AgNO_3$ (180 mg) in ethanol (4 mL) was added, and the mixture was stirred for a few hours. After removing the precipitated AgCl by filtration, the solvent was removed under reduced pressure to obtain $PPh_3Au(NO_3)$ as a white solid (^{31}P-NMR: 27.36 ppm). Finally, to a solution of $PPh_3Au(NO_3)$ (100 mg) in ethanol (5 mL), $NaBH_4$ (0.1 mg) was added and the mixture stirred for 10 minutes. The dark brown solution thus obtained was dried under reduced pressure, and the solid was purified by recrystallization in hexane to obtain [$Au_9(PPh_3)_8$](NO_3) as dark green crystals (^{31}P-NMR: 55.62 ppm)

Synthesis of Chemical Modified Graphene with sulfur functionalities (GOSH). [2] GO (100 mg) was prepared via our adaptation[3] of the Hummers method[4] and dispersed in DMSO (50 ml) via sonication (1 hr). The solution was placed under N_2 and stirred at room temperature before an excess of potassium thioacetate (10 mg) was added. The mixture was then heated to 50 °C for 5.5 hours. After being allowed to cool, HCl (1 M, 5 ml) was added to the GO mixture which was then immediately centrifuged and the solids collected. The solids were washed with acetone (20 minutes, x3), diethyl ether (10 minutes, x2) and distilled water (2 hours, x3) before being dried under vacuum at room temperature to leave a flaky black powder, GO-SH (69.7 mg).

Synthesis of CMG-Au_9 hybrids (GOSH@Au_9). GOSH (30 mg) was dispersed in DMF (30 mL) via sonication for 30 min. [$Au_9(PPh_3)_8$](NO_3) (10 mg) dissolved in ethanol (10 mL) was added to the dispersion, and the mixture was stirred for 1 hr at room temperature. Finally, the solid was collected by centrifugation and thoroughly washed with dichloromethane to remove any unattached [$Au_9(PPh_3)_8$](NO_3).

---

1 a) Cariati, F; Naldini, L. *J.C.S. Dalton.* **1972**, 2286–2287. b)Wen, F.; Englert, U.; Gutrath, B.; Simon, U. *Eur. J. Inorg. Chem.* **2008**, *2008*, 106–111.

2 Thomas, H. R.; Marsden, A. J.; Walker, M.; Wilson, N. R.; Rourke, J. P. *Angew. Chem. Int. Ed. Engl.* **2014**, 7613–7618.

3 Thomas, H. R.; Day, S. P.; Woodruff, W. E.; Valles, C.; Young, R. J; Kinloch, I. A.; Moreley, G. W.; Hanna, J. V; Wilson, N. R.; Rourke, J. P. *Chem. Mater.* **2013**, *25*, 3580-3588.

4 Hummers, W; Offeman, R., *J. Am. Chem. Soc.* **1958,** *80, 1339.*

Crystal structure, distances, and symmetry relationships in $[Au_9(PPh_3)_8](NO_3)$.

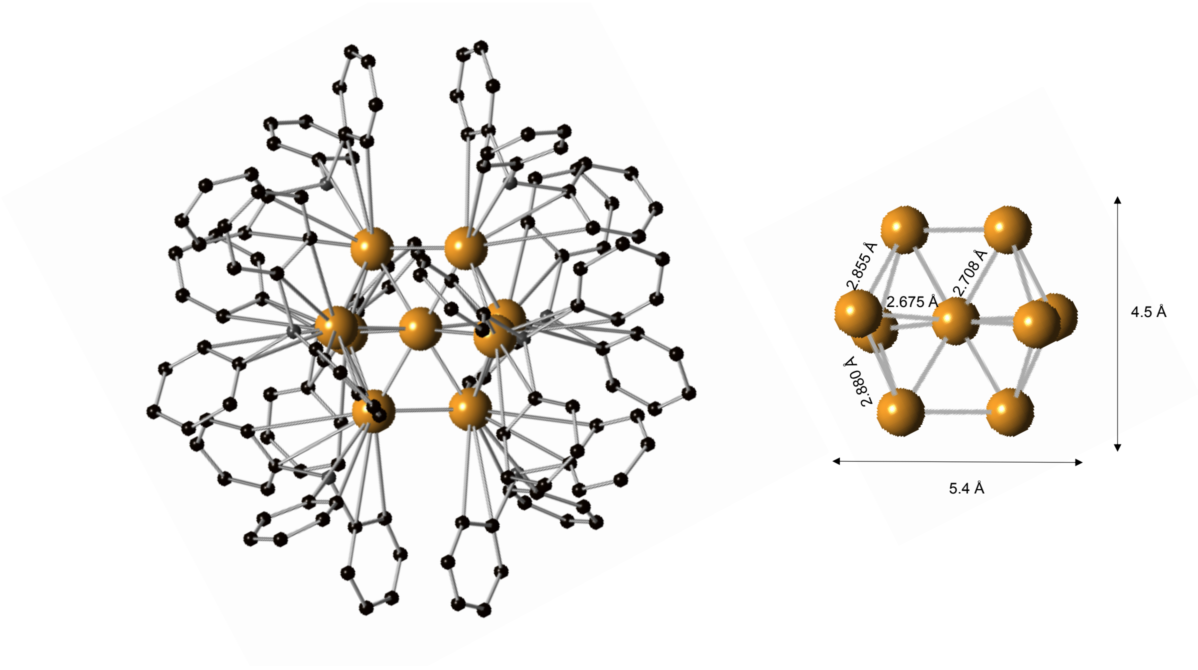

Figure SI.1 The crystal structure of $[Au_9(PPh_3)_8](NO_3)$, with selected distances labelled.

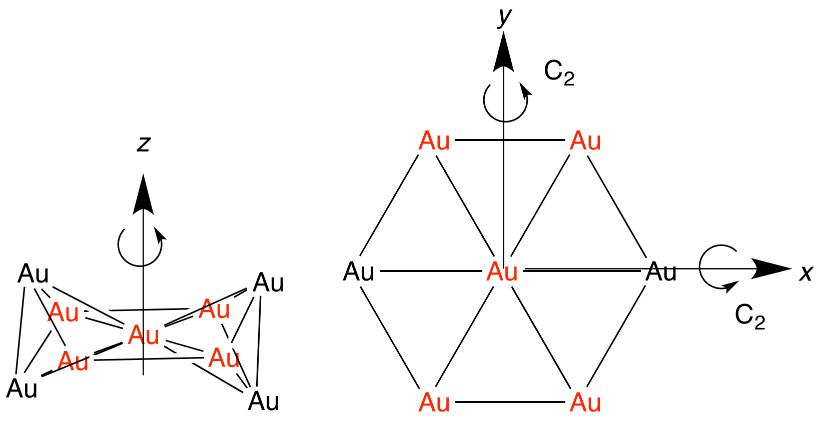

Figure SI.2 Two sketches of the gold cluster: the five red Au define the *x,y* mirror plane of the D_{2h} Au_9 core. A C_2 rotational axis exists perpendicular to the *x,y* plane, and there are two further C_2 axes coincident with the *x,y* coordinate axes.

As shown in the crystal structure, three different types of gold atoms can be observed: (a) the central gold; (b) the four gold atoms at a distance of 2.675 Å from the central gold; and (c) the four gold atoms at a distance of 2.708 Å from the central gold atom (coloured red in SI.2). The central gold is clearly the most inaccessible atom for performing a chemical reaction. Otherwise, gold atoms (b) and (c) are rather similar, although the gold atoms (c) are marginally less sterically impeded, and thus potentially more reactive.

Raman spectroscopy

Raman spectra were collected on a Rainshaw 2000 spectrometer under 514 nm excitation.

Raman spectra of GOSH and GOSH@Au_9 show the G (~1600cm^{-1}) and D (~1360cm^{-1}) bands typical of graphitic materials. We would not expect to see much, if any, difference after anchoring the Au_9 clusters as this does not affect the graphitic nature of the material that is responsible for the Raman response.

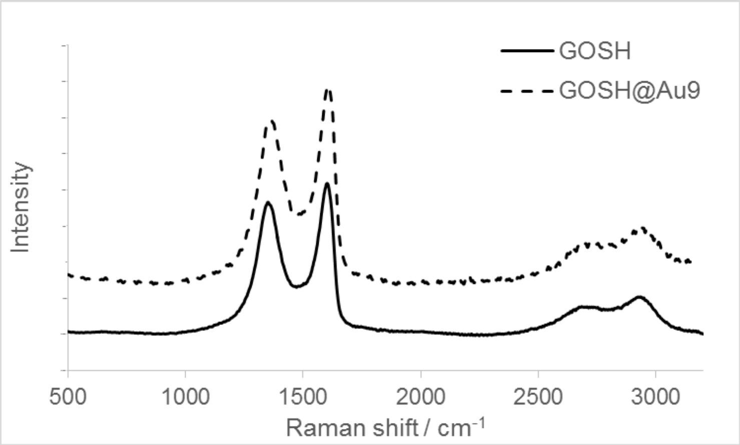

Figure SI.3 Raman spectra of GOSH, (*black solid line*) and GOSH@Au_9 (black dashed line)

X-Ray Photoelectron Spectroscopy (XPS).

The x-ray photoemission spectroscopy (XPS) data were collected at the Warwick Photoemission Facility, University of Warwick, more details of which are available at http://go.warwick.ac.uk/XPS. The samples investigated in this study were deposited on to electrically-conductive carbon tape, mounted on to a sample bar and loaded in to a Kratos Axis Ultra DLD spectrometer with a base pressure of ~ 2 x 10^{-10} mbar.

XPS measurements were performed in the main analysis chamber, with the sample being illuminated using a monochromated Al Kα x-ray source. The measurements were conducted at room temperature and at a take-off angle of 90° with respect to the surface. The core level spectra were recorded using a pass energy of 20 eV (resolution approx. 0.4 eV). The spectrometer work function and binding energy scale were calibrated using the Fermi edge and $3d_{5/2}$ peak recorded from a polycrystalline Ag sample prior to the commencement of the experiments. The data were analysed in the CasaXPS package, using Shirley backgrounds and mixed Gaussian-Lorentzian (Voigt) lineshapes. For compositional analysis, the analyser transmission function has been determined using Ag, Au and Cu foils to determine the detection efficiency across the full binding energy range.

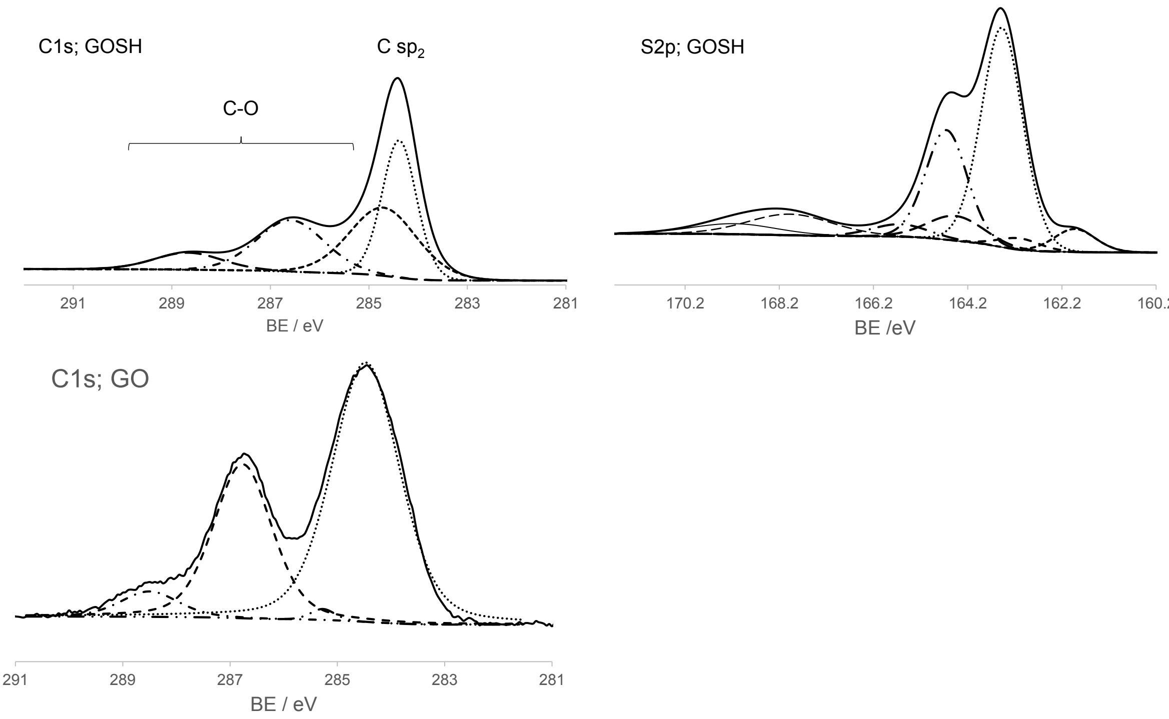

Figure SI.4 (*top*) C1s and S2p XPS core level spectra of GOSH, (*bottom*) C1s XPS spectrum of GO. No signal for sulfur can be found for GO.

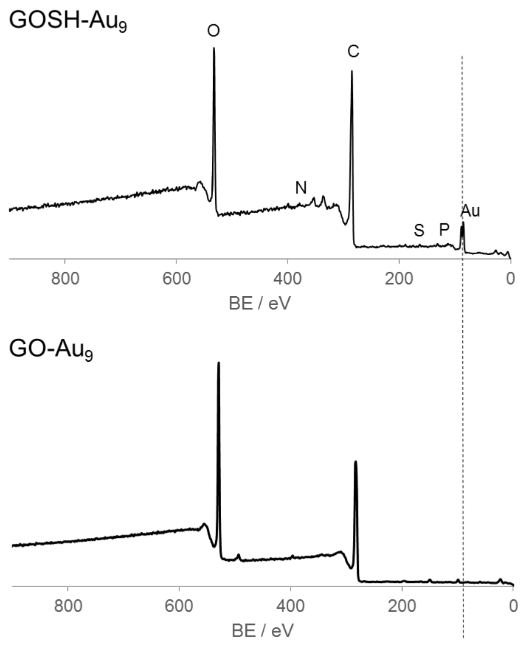

(a)

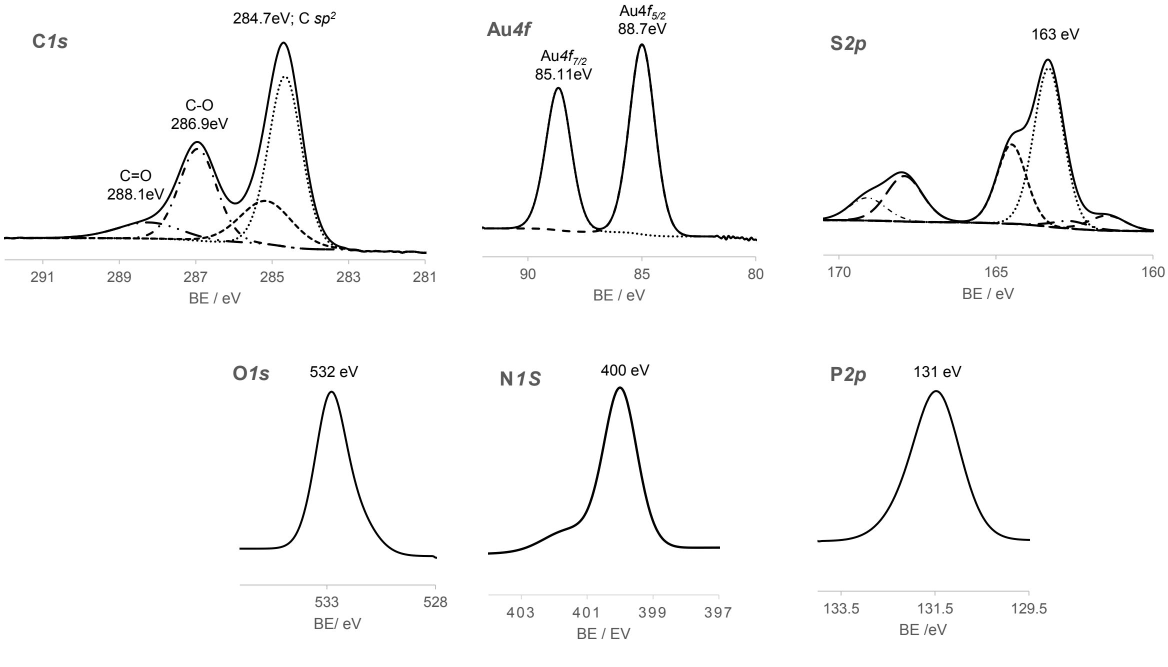

(b)

Figure SI.5 (a) Survey spectra of GOSH@Au_9 and GO after the addition of Au_9. The vertical dashed line highlights the absence of gold in the GO sample. (b) Core level spectra for XPS spectra of GOSH@Au_9.

In S2p XPS spectra, the signal corresponding to S-Au should appear at a binding energy of ~162 eV.[5] Unfortunately, in GOSH the S-S signal also appears at around 162 eV,[2] thus hindering the identification of the S-Au bond by this means.

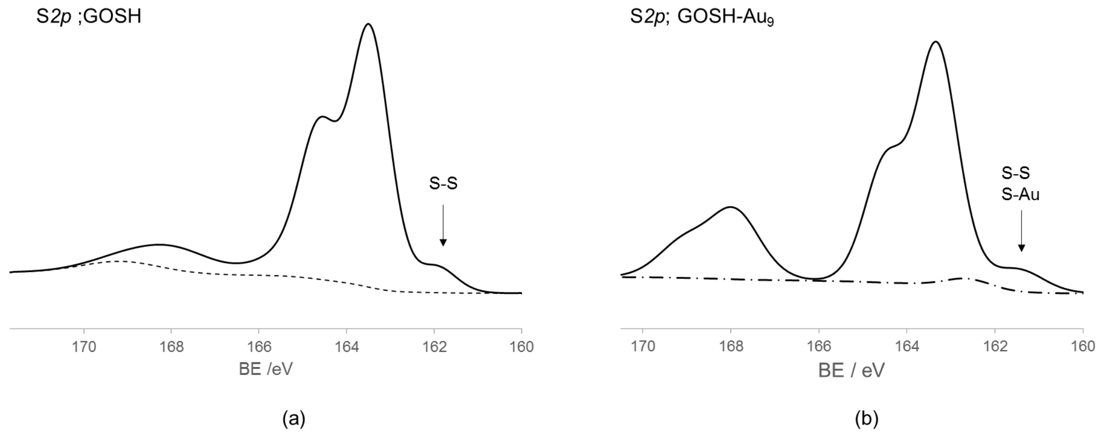

Figure SI.6 S2p spectra of 9(a) GOSH and (b) GOSH@Au_9

From the high resolution XPS scan the following atomic composition can be extracted.

**GOSH@Au_9**					
%C	%O	%Au	%N	%S	%P
72	23.5	1.6	0.7	0.9	1.3

EDX data were recorded on a Zeiss SUPRA 55-VP FEGSEM with an EDAX Genesis analytical system, readings were taken at least four random spots across a sample with the standard deviations from the mean values noted in parentheses.

**% At / GOSH@Au_9**				
%C	%O	%Au	%S	%P
81.3 (±2)	15.9 (±1)	1.2 (±0.2)	0.6 (±0.1)	0.9 (±0.1)

[5] Bourg, M.-C.; Badia, A.; Lennox, B. *J. Phys. Chem. B* **2000**, *104*, 6562-6567

Thermogravimetric analysis of GOSH and GOSH@Au_9.

TGA was recorded on a Mettler-Toledo TGA/DSC1 system at a heating rate of 10 °C /min from 25-800 °C under air.

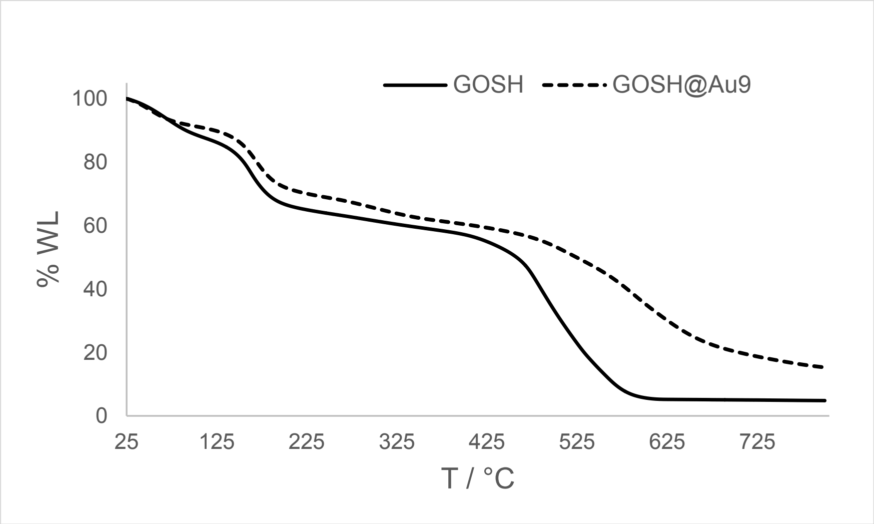

Figure SI.7 TGA traces for GOSH and GOSH@Au_9.

The molar weight of gold is 196.97 g/mol, thus nine gold atoms have a weight of 1772.73 g/mol. The molar weight of carbon is 12 g/mol. Therefore, from the remaining mass in the hybrid (10.5 %), an approximate degree of functionalization can be estimated as follow:

$$(0.895/12)/(0.105/1772.73)=1259$$

This gives a degree of functionalization of approximately one Au_9 per 1260 carbon atoms. Nevertheless, it should be noted that this value is approximated as no consideration is being given to the phosphine ligands and to the oxygen/sulfur groups present across GOSH. To an order of magnitude, we can say approximately one gold cluster per thousand carbon atoms, or 1 atomic % Au.

TEM

For conventional TEM and diffraction a JEOL 2000Fx was used, with an accelerating voltage of 200 kV. Aberration corrected TEM images were collected using a JEOL ARM200F with accelerating voltage of 80 kV and spherical aberration coefficient Cs < 0.001 mm. All images were recorded on Gatan Orius cameras. The minimum dose system (MDS) from JEOL was used to reduce damage caused by the electron beam for atomic resolution images. This involves locating regions of interest with doses below 1 e^-/Å^2/s, before instantly switching to higher magnification and dose (40000e^-/Å^2/s) to record the images. Images were acquired continuously with 0.3 s exposure.

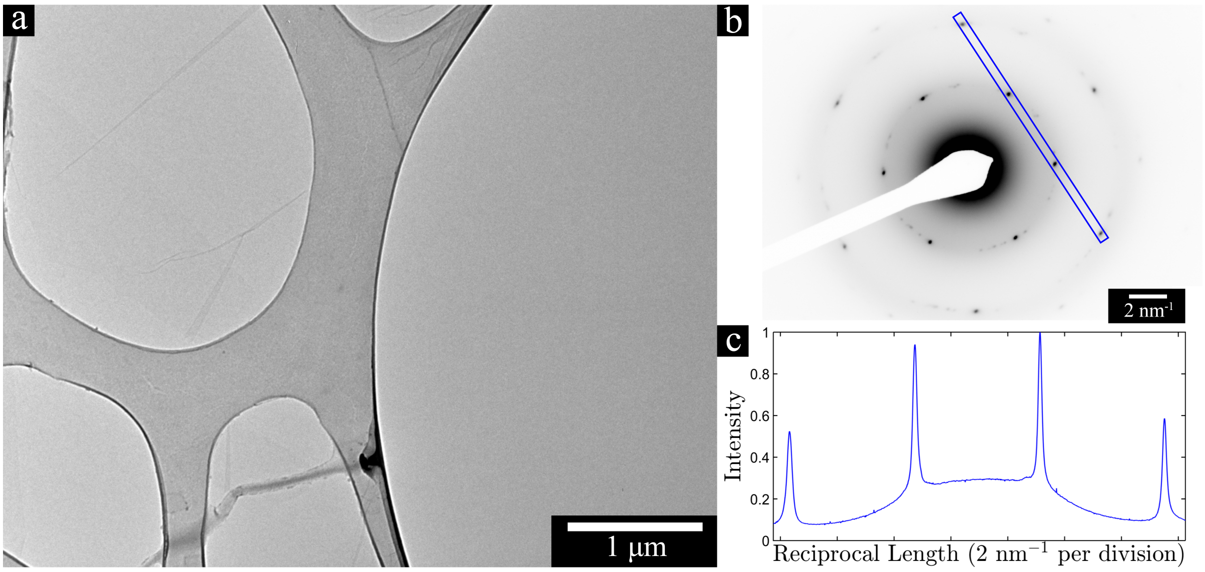

Figure SI.8. a) TEM image showing a sheet of GOSH@Au_9 suspended across a hole in a lacey carbon support. b) A selected area diffraction pattern acquired from a). c) Line profile as marked in the diffraction pattern showing that the outer diffraction peak are less intense than the inner ones, consistent with a monolayer of graphene-like material.

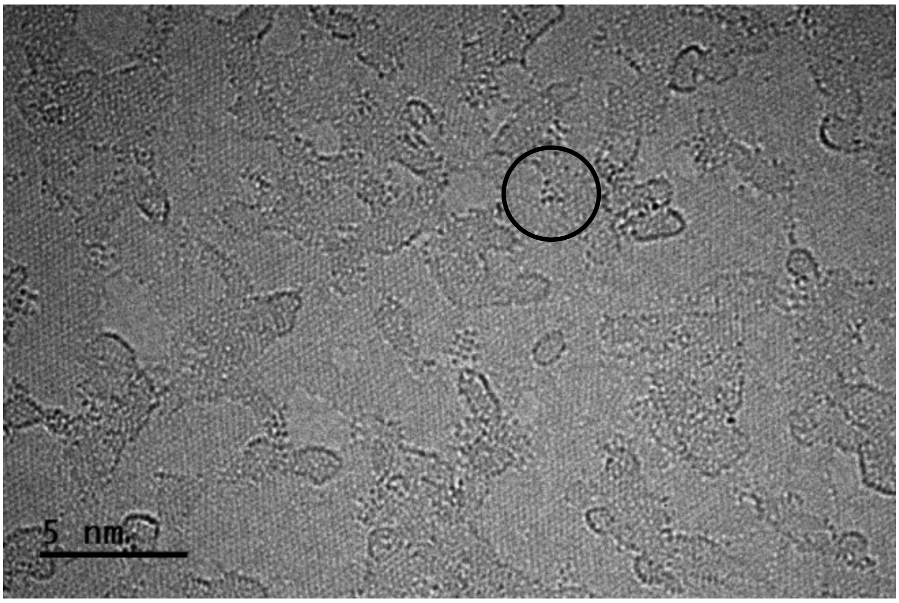

(a)

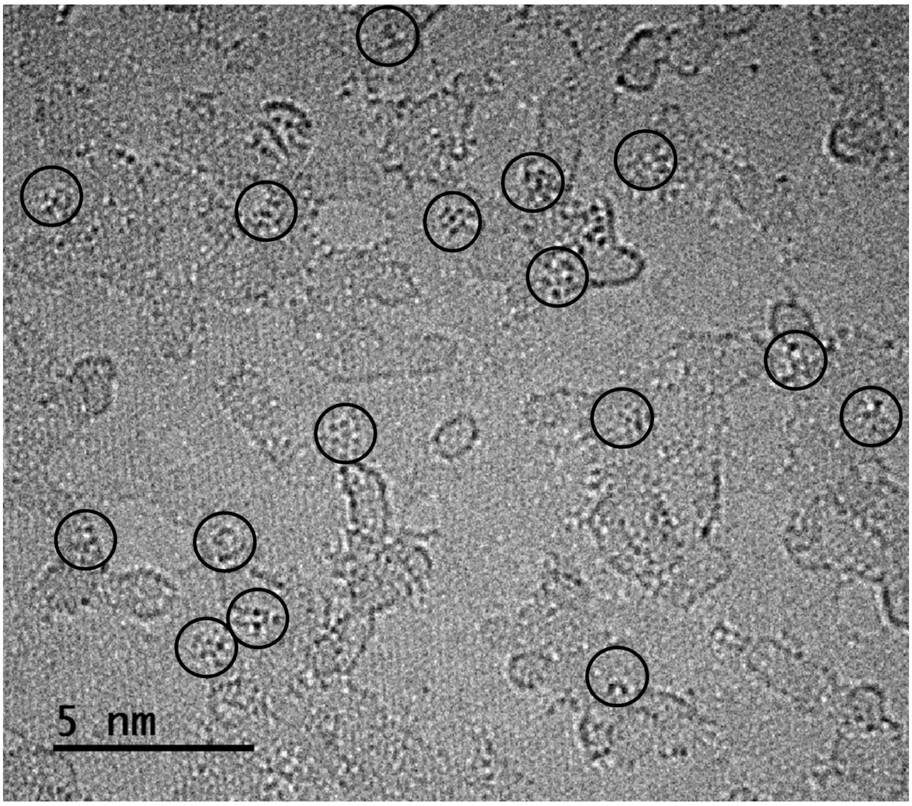

Figure SI.9 ac-TEM image of GOSH@Au_9 acquired at 80 kV. In (a) a black circle highlights the selected Au_9 cluster for performing dynamic studies described in the main text (Figure 2). In (b) some Au_9 clusters are highlighted with a black circle, pointing out the homogeneous distribution of clusters over GOSH

Image simulation

TEM images were simulated using clTEM, an open source, GPU-accelerated multi-slice simulation programme (Dyson, M. A. clTEM: OpenCL TEM/STEM simulation code (2014). URL github.com/Adyson/clTEM). Images were simulated using acceleration voltage 80 kV, Cs = 0.001 mm and 8 nm defocus. CCD detector response was modelled using the method outlined in reference 6

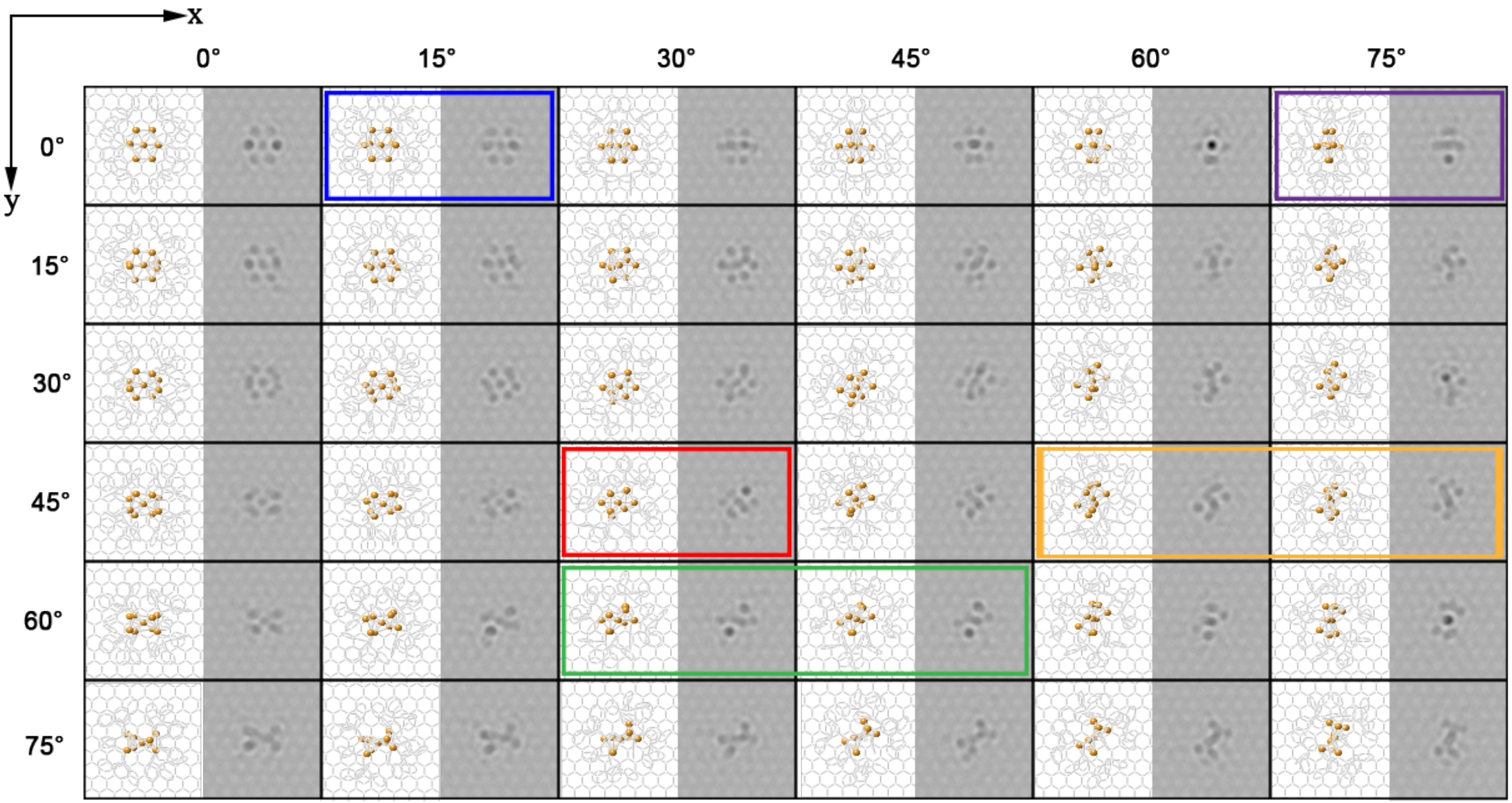

Figure SI.10 Image simulations created by simulating a single $[Au_9(PPh_3)_8]^-$ rotated by 15° intervals about *x* and *y* (axes defined in Figure SI.2). In each column the model is on the left and the corresponding simulation on the right. In coloured squares the approximated orientations found in the dynamical studies (refer to Figure 2) are highlighted as follows: with an orange square (60°, 45°) and (75°, 45°) an approximate orientation matching that of t=0 s; with a blue square (15°, 0°) an approximate orientation matching that of t=0.3 s; with a red square (30°, 45°) an approximate orientation matching that of t=2.1 s; with a green square (30°, 60°) and (45°, 60°) an approximate orientation matching that of t=4.8 s and t=9.3 s; and with a purple square (75°, 0°) an approximate orientation matching that of t=11.1 s.

Metal-Metal distance measurements.

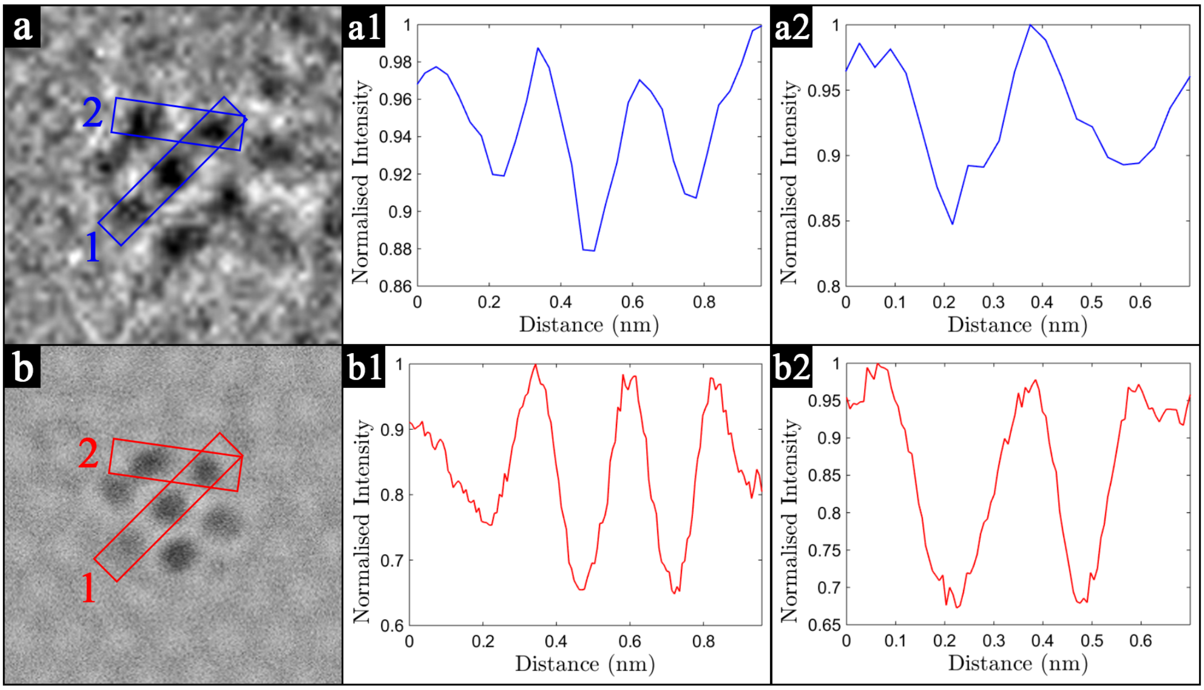

a1: (0.54 ± 0.1 nm), b1: (0.53 ± 0.1 nm)
a2: (0.28 ± 0.1 nm), b2: (0.28 ± 0.1 nm)

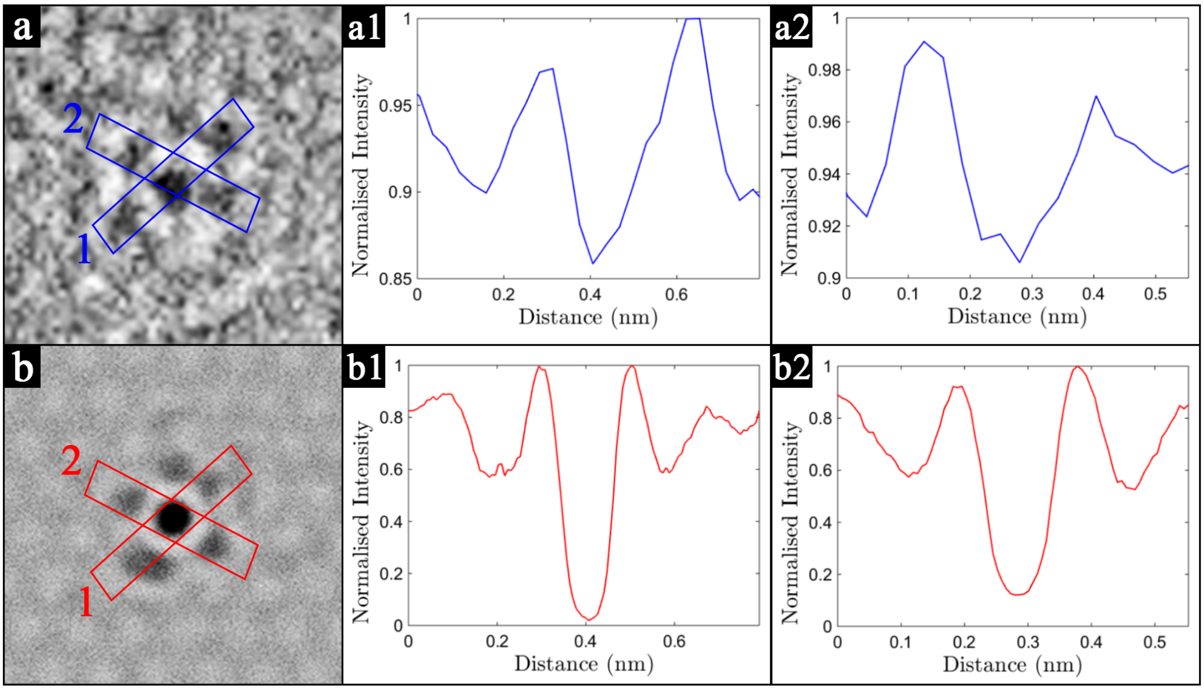

a1: (0.59 ± 0.1 nm), b1: (0.49 ± 0.1 nm)
a2: (0.48 ± 0.1 nm), b2: (0.47 ± 0.1 nm)

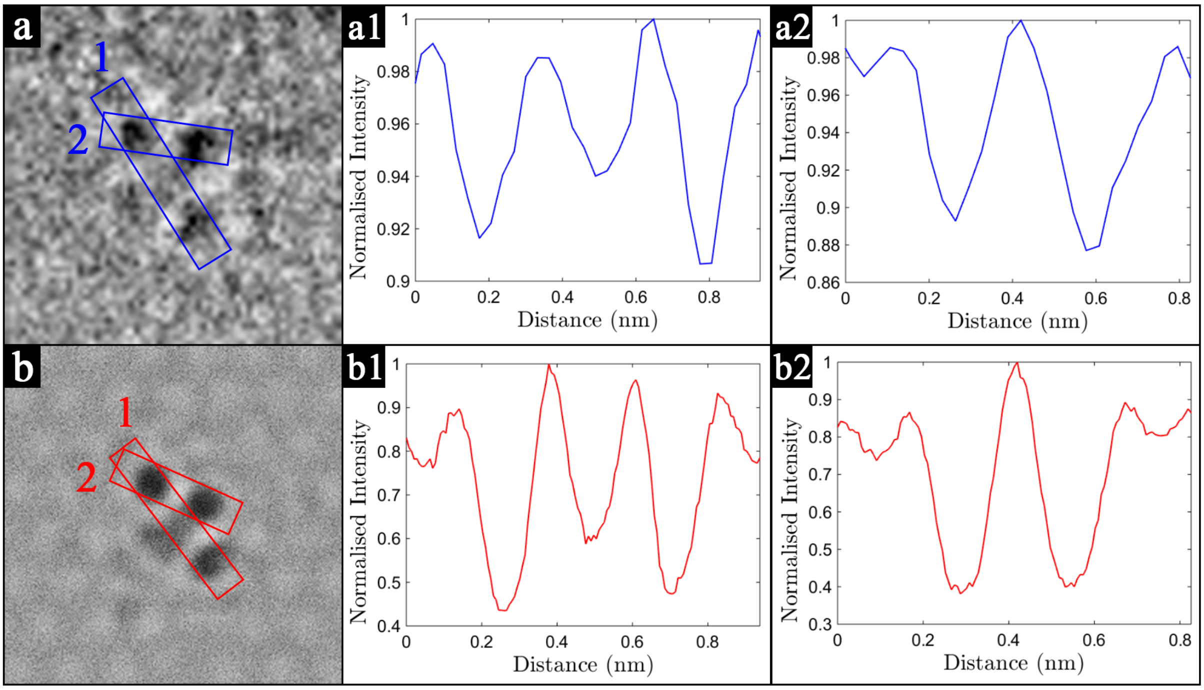

a1: (0.61 ± 0.1 nm), b1: (0.59 ± 0.1 nm)
a2: (0.32 ± 0.1 nm), b2: (0.30 ± 0.1 nm)

Figure SI.11 Metal-metal distances for Au_9 for the three different orientations shown in Figure 1 of the main text. A good correlation is obtained from Au-Au distances as extracted from the ac-TEM images (a) and from the TEM simulation (b). a1/a2 and b1/b2 corresponds to the line profile obtained in the marked areas of each a/b figure. The respective distances are summarized immediately below each simulated ac-TEM image. Note that the simulated images where calibrated according to the known crystal structure and the experimental images were calibrated by comparison between the graphene lattice apparent in the FFT and the known lattice spacing of graphene.

Study of the dynamics

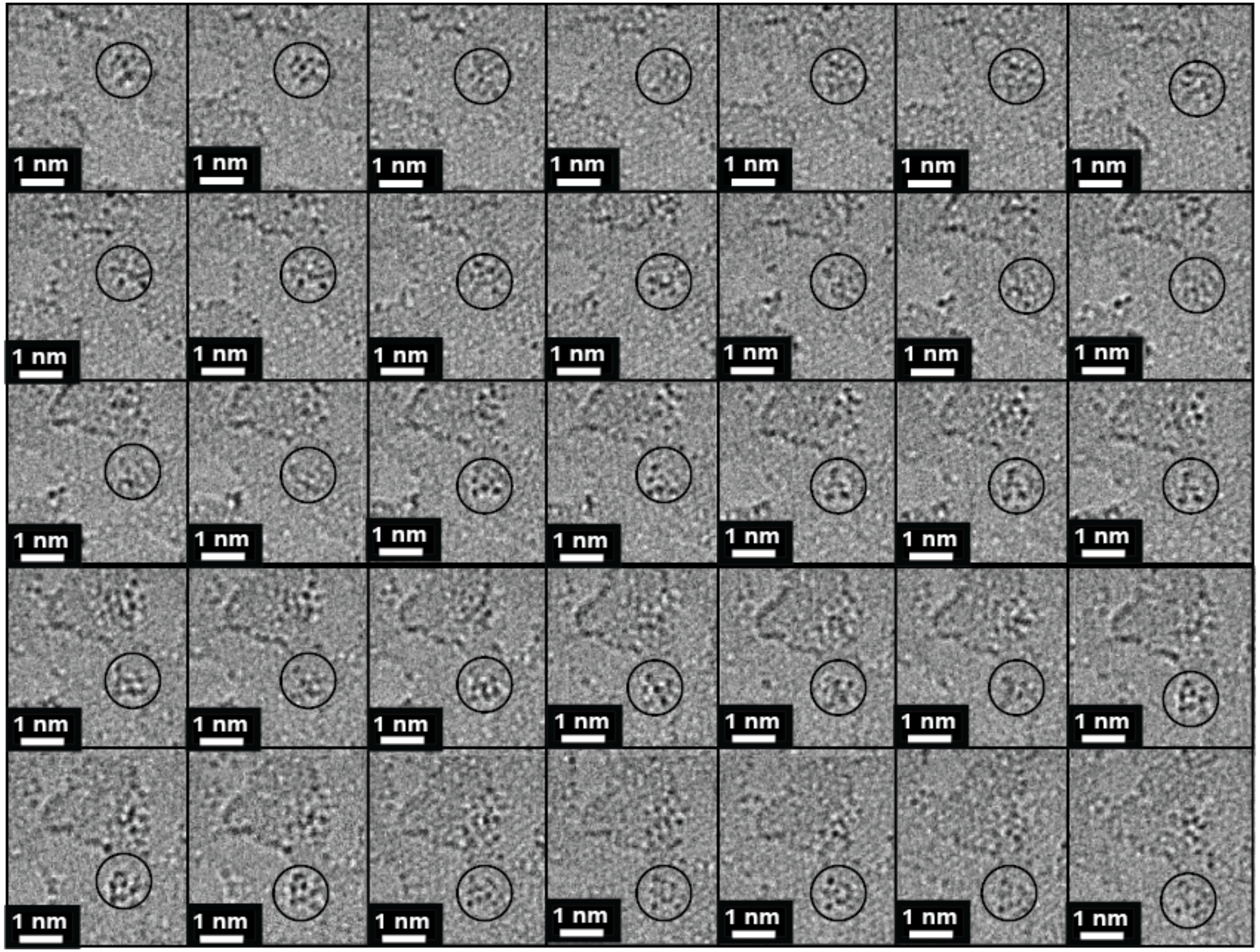

Figure SI.12 Sequence of ac-TEM images of the same Au_9 molecule acquired at 0.3 s intervals over a period of 11.4 seconds.

The images shown in Figure SI.12 were taken from the same region (Figure SI.9a). Comparison with other features in these images shows there is no lateral diffusion of the Au_9. However, the contrast of the cluster changes over time with some frames readily identifiable as a distinct Au_9 orientation through comparison with the image simulation tableau in Figure SI.8. Others that contain more spots could be assigned to the superposition of two metastable states during the image acquisition time for that frame, which complicates accurate identification of the cluster orientation in every frame.
